# A Mediated BOD Biosensor Based on Immobilized *B. Subtilis* on Three-Dimensional Porous Graphene-Polypyrrole Composite

**DOI:** 10.3390/s17112594

**Published:** 2017-11-10

**Authors:** Jingfang Hu, Yueqi Li, Guowei Gao, Shanhong Xia

**Affiliations:** 1 Key Laboratory of Sensor, Beijing Information Science and Technology University, Beijing 100000, China; liyueqi2015@126.com (Y.L.); ggw@bistu.edu.cn (G.G.); 2 State Key Laboratory of Transducer Technology, Institute of Electronics, Chinese Academy of Science, Beijing 100000, China; shxia@mail.ie.ac.cn

**Keywords:** mediated BOD biosensor, immobilized *B. subtilis*, 3D porous graphene-polypyrrole composite, electrochemical measurement

## Abstract

We have developed a novel mediated biochemical oxygen demand (BOD) biosensor based on immobilized *Bacillus subtilis* (*B. subtilis*) on three-dimensional (3D) porous graphene-polypyrrole (rGO-PPy) composite. The 3D porous rGO-PPy composite was prepared using hydrothermal method following with electropolymerization. Then the 3D porous rGO-PPy composite was used as a support for immobilizing negatively charged *B. subtilis* denoted as rGO-PPy-*B* through coordination and electrostatic interaction. Further, the prepared rGO-PPy-*B* was used as a microbial biofilm for establishing a mediated BOD biosensor with ferricyanide as an electronic acceptor. The indirect determination of BOD was performed by electrochemical measuring ferrocyanide generated from a reduced ferricyanide mediator using interdigited ultramicroelectrode array (IUDA) as the working electrode. The experimental results suggested a good linear relationship between the amperometric responses and BOD standard concentrations from 4 to 60 mg/L, with a limit detection of 1.8 mg/L (S/N ≥ 3). The electrochemical measurement of real water samples showed a good agreement with the conventional BOD_5_ method, and the good anti-interference as well as the long-term stability were well demonstrated, indicating that the proposed mediated BOD biosensor in this study holds a potential practical application of real water monitoring.

## 1. Introduction

To date, the problem of organic pollution in river and lake water has been more and more serious in some countries [1]. Biochemical oxygen demand (BOD) is an important parameter for estimating the organic pollution of water [2]. The European Union proposed surface water quality standard on BOD concentration range of the class I~V being 3 mg/L~7 mg/L [3]. People’s Republic of China limited the water quality standard of BOD concentration in surface water should be less than 10 mg/L [4]. And the domestic wastewater discharge standard on BOD concentration range of I~III classed water quality is 10 mg/L~60 mg/L, according to water quality discharge standards in China [5]. So the determination of BOD, especially in low concentration, is significant for real water quality monitoring.

Mediated BOD biosensor has attracted many interests, because it has unique advantages in the BOD fast determination that the added mediator can speed up the biochemical reaction of organic oxidation [6]. Microorganisms suspended in solution are usually used in conventional mediated BOD biosensors [7]. However, tedious and repeated working process such as microorganism cultivation, centrifugation and washing is required before each measurement, which leads to a barrier for BOD fast detection and online environmental monitoring. Microorganism immobilization may provide an effective and economical method in solving the aforementioned problems, and additionally, immobilization can offer other benefits, such as improved metabolic activities, greater stability by minimizing bacterial loss toxic compounds and increased protection against environmental toxicity of pollutants [8]. According to the reported documents, a variety of materials including calcium alginate [9], agar [10], cellulose [11], and polyvinyl alcohol (PVA) [12], and so forth, have been prepared in various geometric formats and used to immobilize microorganisms. In recent years, the three-dimensional (3D) porous structure of graphene is significantly attractive for its biosensor application due to its 3D interconnected porous structure and excellent mechanical stability from the strong π-π stacking of graphene sheets [13,14]. The obtained graphene via hydrothermal reduction can maintain some functional groups such as carboxyl, hydroxyl and epoxy, which are easy to make functionalized modification for sensor development [15].The use of 3D porous graphene as a support for the immobilization of enzymes and whole cells has largely emerged [16,17,18], but little attention was paid to using 3D porous graphene for microorganism immobilization.

The microorganism of *Bacillus subtilis* (*B. subtilis*) is an eco-friendly bacteria, and has been well studied as a microbial biofilm for BOD measurement [19]. *B. subtilis* has negatively charged lipids, and its p*K*a values were reported to be 4.8, 6.9 and 9.4, which likely correspond to amino, phosphate, and hydroxyl sites [20]. The p*K*a values as well as reported metal sorption evidence showed that anionic groups dominate the surface in the neutral pH region [21]. Considering the enriched functional groups and negative charges on *B. subtilis* bacterial membrane, it is reasonable to question whether such a material with corresponding ligand bonds and positive charges can be used as an ideal support for *B. subtilis* immobilization through coordination and electrostatic interactions [22].

Polypyrrole (PPy) is one of the most widely used materials for sensor and biosensor development because of its good chemical stability and biocompatibility [23]. Due to its special polymeric matrix structure, PPy can be easily functionalized and controlled to immobilize various biomolecules, such as enzymes and DNA through adsorption, entrapment, covalent binding, cross-linking or a combination of these techniques [24]. Recently, PPy has been found to be used in immobilization of viable bacilliform bacteria thanks to its positive charges during electropolymerization and the bacilliform bacteria’s own carried negative charges [25].

In this work, 3D porous graphene-polypyrrole (rGO-PPy) composite was synthesized by two steps for *B. subtilis* immobilization. Firstly, hydrothermal method was employed to prepare graphene-pyrrole (rGO-Py) hydrogels with large pores. Herein, pyrrole was added in graphene oxide (GO) dispersion in order to hinder the assembly of rGO (reduced graphene oxide, also named graphene) sheets and adjust the pore size of resultant rGO hydrogels, and the obtained graphene can coordinate with the groups of amion and hydroxyl on *B. subtilis*. Secondly, rGO-PPycomposite was situ-synthesized via electrochemical oxidation of rGO-Py. PPy layer holds a large amount of positive charges during electrochemical oxidation of Py, and *B. subtilis* with negative charges can be expected to be adsorbed on the PPy matrix to maintain charge neutrality. To the best of our knowledge, up to now, there has been no report about the immobilization of microorganisms on rGO-PPy composite. The immobilized *B. subtilis* on rGO-PPy composite was denoted as rGO-PPy-*B*, which was placed into a small bioreactor as a microbial biofilm for biocatalytic degradation of organic substances in water sample. Ferricyanide, a commonly used artificial electron acceptor for rapid BOD analysis, was added in water samples as a redox mediator to replace oxygen in the respiratory pathway of *B. subtilis*. As well, interdigited ultramicroelectrode array (IUDA) was fabricated by MEMS (micro-electro-mechanical systems) technology for electrochemical determination of mediated BOD. Finally, the electrochemical characteristics and high performance of the resultant mediated BOD biosensor with respect to linear range, the detection limit, reproducibility, selectivity and stability was investigated in detail. 

## 2. Experiment Section

### 2.1. Materials and Reagents

10 mL Graphene oxide (GO) aqueous dispersion (1 mg/mL) was prepared with 10 mg GO powder in 10 mL deionized water and supersonically vibrated for 1 h. Dried cells of *B. subtilis* were acquired from China General Microbiolgical Culture Collection Center. GO powder, acridine orange (AO) were purchased from Sigma & Aldrich (Buchs, Switzerland). Py monomer, K_3_Fe(CN)_6_, glucose, glutamic acid, Na_2_HPO_4_, K_2_HPO_4_, H_2_SO_4_, NaCl, petone and beef extract were obtained from Sinopharm Chemical Reagent Beijing Co. Ltd. (Beijing, China). Phosphate buffer solution (PBS, 5 mM, pH 7.0) was prepared with 0.68 g K_2_HPO_4_ and 1.34 g Na_2_HPO_4_. The BOD standard solution with a concentration of 2500 mg/L was prepared with 1.705 g glucose and 1.705 g glutamic acid dissolved in 5 mM PBS solution according to procedures described in the HJ/T 86-2002 standard method [26]. BOD solutions with other concentrations were prepared by appropriate dilution of the 2500 mg/L BOD standard solution with 5 mM PBS solution. Unless otherwise stated, all chemical solutions were of analytical grade and prepared with deionized water.

### 2.2. Bacterial Cultivation

*B. subtilis* cells were firstly inoculated from slant into the liquid medium solution and incubated at 37 °C for 6 h with shaking at 150 rpm. The liquid medium solution contained 10 g peptone, 3 g beef exact and 5 g NaCl, and pH was adjusted to 7.0. After cultivation, the cell concentration reached 3.6 × 10^5^ CFU/mL. The 0.2 mL, 1 mL, 5 mL, 10 mL and 20 mL cell suspensions were, respectively, inoculated into 200 mL new liquid medium with 12 h cultivation to reach a stationary phase [27] and their cell concentration of 0.2 × 10^7^ CFU/mL, 1.1 × 10^7^ CFU/mL, 5.4 × 10^7^ CFU/mL, 9.8 × 10^7^ CFU/mL and 19.7 × 10^7^ CFU/mL were obtained by plate counting method. Then the cultivated cell suspensions were harvested by centrifugation at 7000 rcf for 20 min at room temperature. Finally the cells were washed thrice in PBS (5 mM, pH 7.0) and stored at 4 °C.

### 2.3. Preparation of 3D Porous rGO-PPy Composite

**Figure 1:**
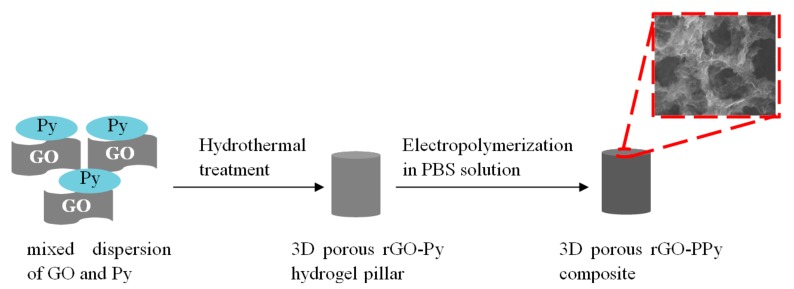
The scheme of preparation of the three-dimensional (3D) porous graphene-polypyrrole (rGO-PPy) composite.

10 mL Graphene oxide (GO) aqueous dispersion (1 mg/mL) was prepared with 10 mg GO powder in 10 mL deionized water and supersonically vibrated for 1 h. Then the 10 mL GO aqueous dispersion was mixed with 500 µL pyrrole monomer under stirring. The mixture was then sealed in a 20 mL Teflon-lined autoclave at 180 °C for 12 h. After cooling down to room temperature, 3D porous rGO-Py hydrogel pillar was obtained. Finally, the 3D porous rGO-Py hydrogel pillar was directly used as electrode and immersed in PBS solution without any additional Py monomer. A constant potential of 0.8 V was applied on the rGO-Py hydrogel pillar for electropolymerizationof Py for 30 min and 3D porous rGO-PPycomposite was in situsynthesized. The scheme of preparation of the 3D porous rGO-PPycomposite was illustrated in Figure 1.

### 2.4. Microorganisms Immobilization and Bioreactor Fabrication

The harvested *B. subtilis* cell suspensions were pulled into 200 mL liquid medium solutions to their original concentrations, respectively. Then the prepared 3D porous rGO-PPy composite was immersed in the *B. subtilis* solution and shacked under 37 °C at 100 rpm for 6 h, 12 h and 24 h. After culture, the 3D porous rGO-PPy immobilized with *B. subtilis* (rGO-PPy-*B*) was obtained as a microbial biofilm. The microbial biofilm was filled into a 2.0 mL Eppendorf tube to fabricate a small bioreactor and stored in 5 mM PBS solution (pH 7.0).

### 2.5. Microscopic Observations

The morphology of the as prepared composites were characterized by scanning electron microscopy (SEM, JSM-6390LV, JEOL, Tokyo, Japan) and transmission electron microscopy (TEM, JEM-1200EX, JEOL, Tokyo, Japan). X-ray diffraction (XRD) patterns were obtained using a XHHO3 X-ray fluorescence spectrometer (Spectro X-sort, SPECTRO Analytical Instruments GmbH, Kleve, Germany). Fourier-transform infrared spectroscopy (FTIR) spectra were recorded on a Nicolet iS5 Fourier-transforminfrared spectrophotometer (Thermofisher, Waltham, MA, USA) using KBr pellet. The rGO-PPy-*B* microbial biofilm were stained with acridine orange (AO), according to the manufacturer’s instructions. Then the rGO-PPy-*B* microbial biofilmwas washed with deionized water and examined by confocal laser scanning microscopy (CLSM, FV3000, Olympus, Tokyo, Japan).

### 2.6. Electrochemical Experiments

All electrochemical experiments were carried out with the Reference 600 workstation (Gamry Instruments, Warminster, PA, USA) and performed in a standard three-electrode system, where a segment of an interdigited ultramicroelectrode array (IUDA) as the working electrode, the other segment of IUDA microelectrode as the counter electrode, and Ag/AgCl electrode as the reference electrode. The used IUDA microelectrode (*w_e_* = *w_g_* = 10 µm) was fabricated by MEMS technology as our previous report [28].Linear sweep voltammetry (LSV) was used to characterization of the effectiveness of rGO-PPy-*B* microbial biofilm for BOD measurement, scanning from −0.2~0.6 V with the scan rate of 50 mV/s. Chronoamperometry was used to BOD measurement at the potential of 0.6 V for 20 s.

### 2.7. BOD Measurement Procedures

2 mL sample solution for incubation, prepared with the mixture of potassium ferricyanide solution and BOD standard solution, was pulled into the bioreactor. Endogenous control solutions were prepared by adding 5 mM PBS in place of the test BOD substrate. Before measurement, the mixture was encapsulated into the bioreactor and incubated at 37 °C for 15 min. The temperature was controlled by water bath. After incubation, the sample solution was pumped out by syringe and then used for analysis of microbially accumulated ferrocyanide at 25 °C.A potential of 0.6 V was applied to the working electrode of IUDA. The chronoamperometric time for BOD detection was set as 20 s when the signal was stable. The IUDA was pretreated by ultrasonic cleaning in acetone, in ethanol, and in deionized water for 5 min, respectively. Then the electrode was also electrochemically cleaned in 5 mM H_2_SO_4_ by cycling the electrode potential from 0 to 1.5 V until a reproducible voltammogram was obtained.

### 2.8. Measurement of Real Water

Mixture solution of 20 mM ferricyanide (final concentration) and real water sample were added into the bioreactor. The mixture solution was incubated at 37 °C for 15 min. Chronoamperometric measurement of BOD in real water samples after a calibration curve obtained by measurement of BOD standard solutions, and the BOD concentrations of real water samples were calculated according to the calibration curve. The three real water samples were collected from lake water, river water and domestic waste water around Beijing city.

## 3. Results and Discussion

### 3.1. The Detection Mechanism of the Mediated BOD Biosensor

**Figure 2:**
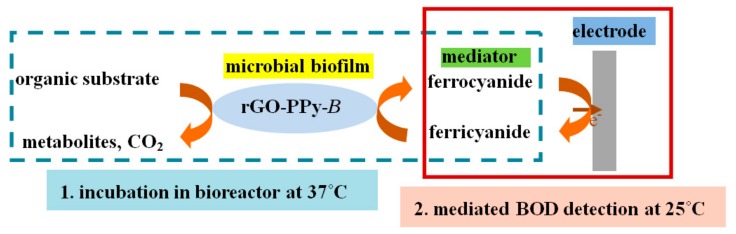
Schematic detection mechanism of the mediated biochemical oxygen demand (BOD) biosensor.

The biosensor for BOD measurement using mediator replaced of oxygen are called mediated BOD biosensor [29]. Ferricyanide was first reported by Pasco et al. [30] for BOD fast detection in 2000, which differs from the standard BOD_5_ method through a significant reduction in incubation time. The incubation time can be reduced greatly due to the high solubility (~10,000 times more soluble than oxygen), which is attractive for the development of rapid BOD biosensors. The proposed mediated BOD biosensor in this study incorporates the ferricyanide ion as a mediator that acts as an artificial electron acceptor, replacing oxygen as the terminal electron acceptor, in the microbial oxidation of organic substrates. Before measurement, it was taken first that the water sample should be incubated for 15 min at 37 °C with rGO-PPy-*B* microbial biofilm and ferricyanide mediator in a small bioreactor. During the incubation, the biochemical reaction of microbial oxidation of organic substance conducted as the following equation (1), that ferrocyanide was generated from ferricyanide reduction. Then the incubation solution was centrifuged into electrochemical cell at room temperature of 25 °C for electrochemical detection of ferrocyanide (mediated BOD).The BOD detection mechanism of the proposed mediated BOD biosensor is illustrated with schematic form as shown in Figure 2.

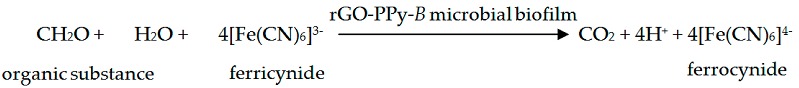
(1)

### 3.2. Characterization of 3D rGO-PPy Composite

**Figure 3:**
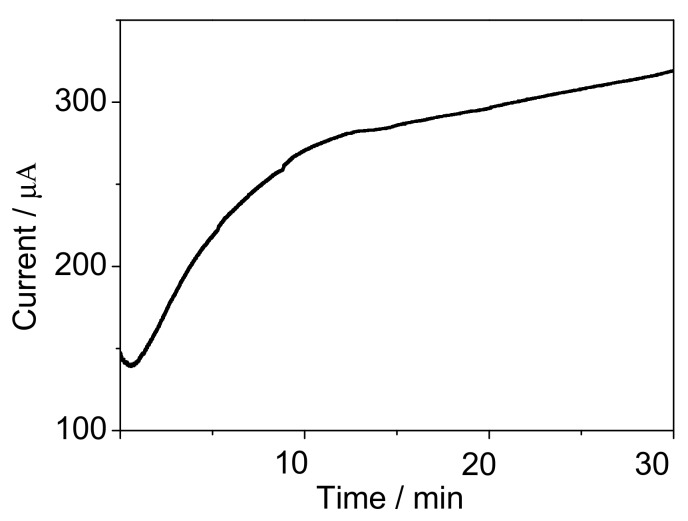
The chronoamperometry curve for electropolymerization of rGO-PPy at the potential of 0.8 V.

**Figure 4:**
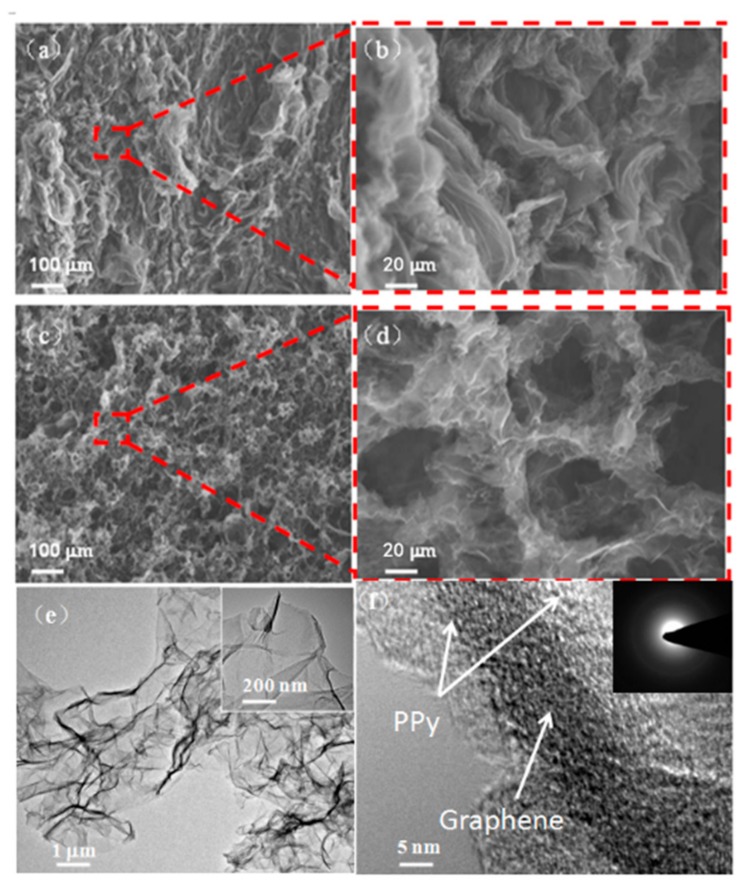
Scanning electron microscopy (SEM) and transmission electron microscopy (TEM) images of the interior microstructures of rGO-PPy composite hydrogels. (**a**,**b**) SEM images of the rGO-PPy obtained from Py concentration of 0.1 mM. (**c**,**d**) SEM images of the rGO-PPy obtained from Py concentration of 1 mM. (**e**,**f**) TEM images of the resulting rGO-PPy. The inset in (**e**) is the enlarged view of the edge. The inset in (**f**) is the selected area electron diffraction pattern of (**e**).

The electrochemical characterization of the PPy situ polymerization process is shown in Figure 3. It was found that the current value gradually increased as the time prolonging under the constant potential of 0.8 V, which suggested that Py in rGO-Py had been oxidized to PPy. Py in the GO dispersion can prevent the self-stacked behavior of rGO sheets during 3D interconnected porous structure formation [31]. Indeed, further control experiments confirmed that the added concentrations of Py can be used to adjust the pore size of the 3D rGO structure. The as-prepared rGO-PPy composite were characterized by SEM after a vacuum freeze-drying treatment. The SEM images of Figure 4a–d illustrated that the pore size increased as the Py concentration increasing from 0.1 mM to 1 mM, the reason forwhich can be speculated as follows. It has been known that during the hydrothermal treatment of pure GO aqueous dispersion, rGO sheets would be self-stacked owing to the gradually increased hydrophobic portion and π-π interactions between rGO sheets, and the stronger attractive interaction will result in a 3D rGO structure with smaller pore size [32]. The additional Py monomer, which has a typical conjugated structure with electron-rich N atom, could easily attach on the surface and galleries of GO sheets through H-banding or π-π interactions and effectively prevent the self-stacked behavior of rGO sheets during heat treatment. When a low concentration of Py was used, only a small amount of Py would attach on rGO sheets and the interactions between rGO sheets was still strong, then resulting in smaller pores. When the higher concentration of Py was used, the larger amount of Py could be adsorbed on rGO sheets and the increased electrostatic repulsion between rGO sheets would partially offset the hydrophobic and π-π interactions between rGO sheets, accordingly leading to enlarged pores. As shown in Figure 4d, when the Py concentration was increased up to 1 mM, the composite has well-defined 3D interconnected porous microstructures. The uniform coverage of PPy on the whole surface of rGO sheets and no aggregated particles were observed in Figure 4e. The high resolution TEM image as shown in Figure 4f further revealed that the electropolymerizedPPy layers covered the graphene sheets, and the electron diffraction spots of graphene (inset in Figure 4f) almost disappeared due to the full coating of graphene sheets with PPy. It is worth noting that the pore diameters of about 20 µm were obtained at 1mM Py, which was thought to be an ideal pore size for *B. subtilis* cells growth [33].

**Figure 5:**
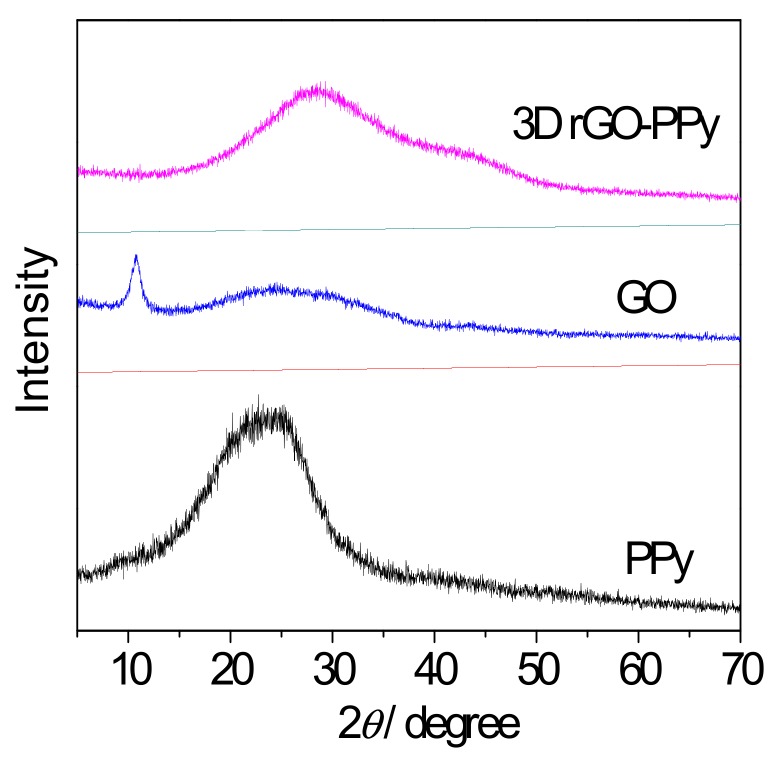
X-ray diffraction (XRD) patterns of PPy, graphene oxide (GO) and 3D rGO-PPy.

**Figure 6:**
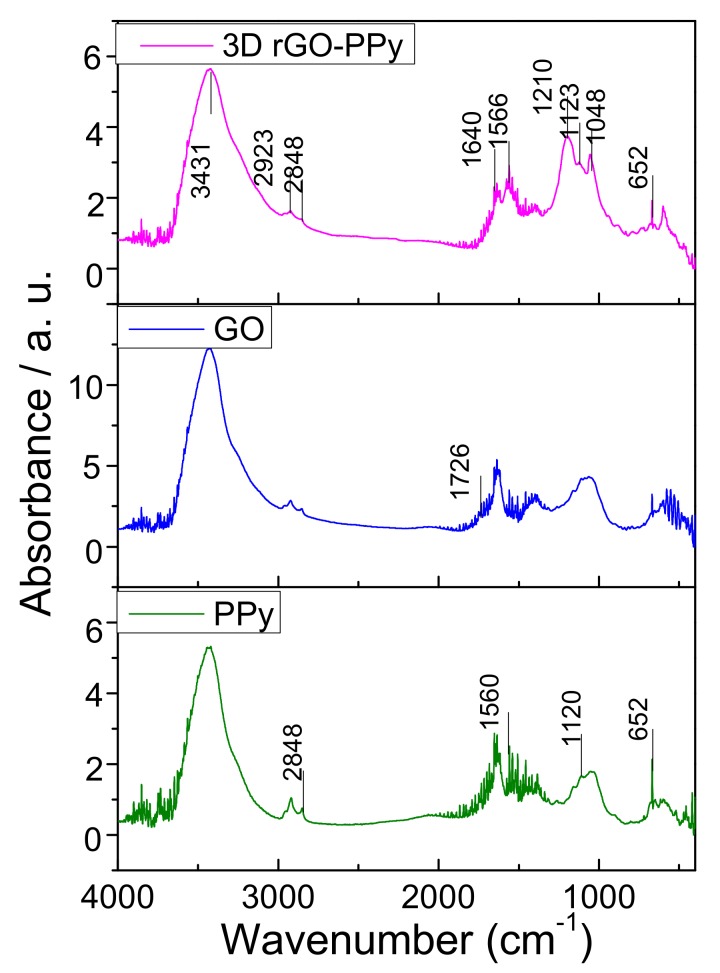
Fourier-transform infrared spectroscopy (FTIR) spectra of PPy, GO and 3D rGO-PPy.

The components of the obtained 3D GO-PPy composite was further confirmed by XRD and FTIR measurement. Figure 5 displayed the XRD patterns of PPy, GO and 3D rGO-PPy, respectively. As can be seen in case of initial GO, a sharp peak centered at 2*θ* = 11°, which is the typical GO crystalline structure and disappeared in case of prepared 3D rGO/PPy composite. This indicates that during hydrothermal process the GO was successfully reduced to rGO by hydrogen ion (H^+^) from water molecules under the influence of high temperature and high pressure, and the ordering characteristic of GO nanosheet was destructed during the reduction process. Moreover, in case of pure electropolymerized PPy, a broad peak was found at 2*θ* = 23° representing the characteristic peak of amorphous structure [34], but shifted to 2*θ* = 28° for the prepared 3D rGO/PPy composite. This suggests that PPy was really in situ synthesized by electropolymerization of 3D rGO/Py, while the *π*-*π* stacking interaction between PPy chains and rGO nanosheets caused the generation of diffraction peak shifted. Figure 6 shows the FTIR spectra of the PPy, GO and 3D rGO/PPy. For 3D rGO/PPy, the absorption strengths compared with the characteristic absorption bands of GO are obviously reduced and even disappeared at 1726 cm^−1^, and corresponded to C=O stretching vibration [35]. The peak at 3431 cm^−1^ is assigned to the O-H stretching vibration, and the bands at 2923 cm^−1^ is associated with the strong relation between carboxyl group and hydroxyl group. The peaks located at 1640 cm^−1^, 1210 cm^−1^ and 1048 cm^−1^ are assigned to C-OH bending vibration, hydroxyl stretching vibration, and epoxy stretching vibration [36]. These results indicates that GO was successfully reduced into rGO during the hydrothermal reaction, but some functional groups such as hydroxyl, carboxyl and epoxy, are still maintained on rGO. The new absorption peaks at 1566 cm^−1^ and 1123 cm^−1^ could be caused by PPy ring-stretching vibration of C-N, and their little shifts are associated with the *π*-*π* interaction between rGO and PPy. In addition, the characteristic absorption bands of PPy at 2848 cm^−1^ and 652 cm^−1^ [37] further demonstrate that PPy has been in situ electropolymerized on rGO sheets.

### 3.3. Characterization of rGO-PPy-B Microbial Biofilm

**Figure 7:**
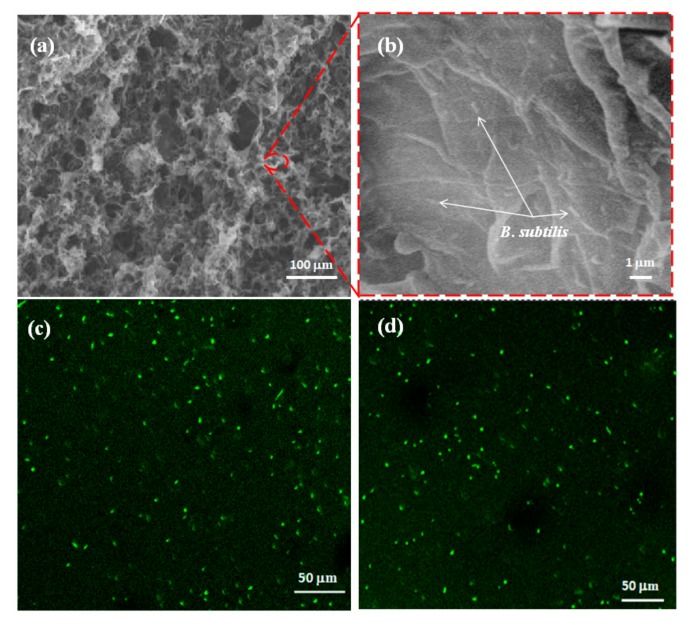
SEM and confocal laser scanning microscopy (CLSM) images of rGO-PPy-*B* microbial biofilm. (**a**) Typical SEM image of the interior microstructure of rGO-PPy-*B* microbial biofilm; (**b**) the magnified SEM image of (**a**); CLSM images of rGO-PPy-*B* microbial biofilm collected at the surface (**c**) and 6 mm depth (**d**).

To investigate the possibility of a microbial biofilm, the proposed 3D porous rGO-PPy composite (by adding 1 mM Py) was used for microorganism immobilization via culture in *B. subtilis* bacteria solution for 24 h. The resulted microbial biofilm of 3D rGO-PPy composite immobilized with *B. subtilis* was represented as rGO-PPy-*B* microbial biofilm. Then the as-prepared rGO-PPy-*B* microbial biofilm was characterized by SEM and CLSM studies. The cross-section SEM images of rGO-PPy-*B* are shown in Figure 7a,b. It is found that the rGO-PPy-*B* still presented 3D porous structure after microorganism immobilization, and rod-shaped bacteria can be observed on the surface, which suggests that *B. subtilis* were successfully immobilized on 3D rGO-PPy composite. Further, the rGO-PPy-*B* microbial biofilm was stained by acridine orange (AO) for fluorescence imaging. AO is widely used as a marker for distinguishing between dead and living cells [38]. As displayed in Figure 7c,d of the representative CLSM images, the significant green fluorescence were observed at both the surface and the inside of rGO-PPy-*B* microbial biofilm, indicating that the immobilized *B. subtilis* were viable and the porous penetration structure was beneficial for more bacteria to be immobilized in the microbial biofilm. So the above results demonstrated that the 3D porous rGO-PPy composite was an ideal supportive substrate for *B. subtilis* immobilization.

**Figure 8:**
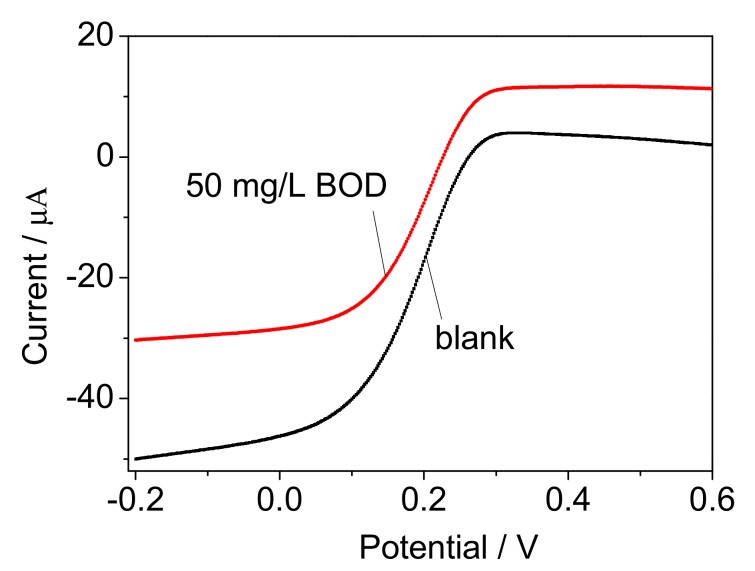
Linear sweep voltammetry (LSV) curves recorded at the interdigited ultramicroelectrode array (IUDA) for 50 mg/L BOD and blank solution after 15 min incubation with the rGO-PPy-*B* microbial biofilm in a bioreactor (both the 50 mg/L BOD solution and blank solution have 20 mM ferricyanide). Scan rate: 50 mV/s; each curve was the average of three replicate measurement.

Figure 8 showed the LSV curves for 50 mg/L BOD and blank solution (5 mM PBS and 20 mL ferricyanide, pH 7.0) after 15 min incubation with the prepared rGO-PPy-*B* microbial biofilm in bioreactor. The added concentration of ferricyanide mediator in water samples and blank solution were both 20 mM. It is found that the amperometric response of 50 mg/L BOD obviously increased compared with the blank solution, suggesting that the rGO-PPy-*B* microbial biofilm had the effective respiration for biodegradation of organic substance and could be potential for further BOD analysis.

**Figure 9:**
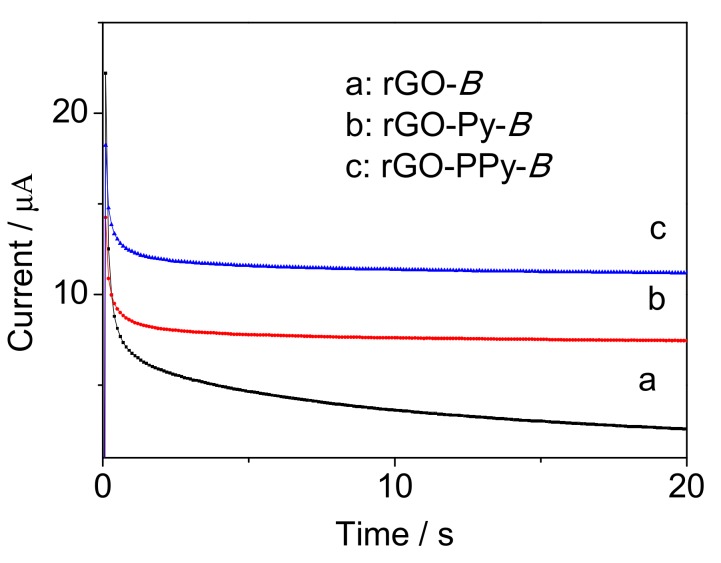
The chronoamperometry signals measured for 50 mg/L BOD using the prepared microbial biofilms of rGO-*B* (a), rGO-Py-*B* (b) and rGO-PPy-*B* (c), respectively.

Furthermore, the wet weight method was used to compare the amount of immobilized *B. subtilis* on rGO, rGO-Py and rGO-PPy. After immobilization under the same condition, rGO-*B*, rGO-Py-*B* and rGO-PPy-*B* were all measured by electronic balance to obtain wet weight. As shown in Table 1, the immobilized cells on rGO-Py are more than on rGO, which further demonstrates that Py can enlarge the pore size of rGO hydrogel and higher loading capability would thus be obtained. Additionally, more cells on rGO-PPy than rGO-Py suggests that positively charged PPy can improve the adsorption capacity for negatively charged *B. subtilis* cells. Then electrochemical test was carried out by comparing rGO-*B*, rGO-Py-*B* and rGO-PPy-*B* as microbial biofilm in bioreactor for BOD determination with the same concentration of 50 mg/L BOD. As shown in Figure 9, the signal from rGO-PPy-*B* was 1.5 times higher than rGO-Py-*B* and 4.2 times higher than rGO-*B*. This result further demonstrated that rGO-PPy was the most ideal support material for *B. subtilis* immobilization than rGO and rGO-Py.

### 3.4. Optimization of Experimental Conditions

**Figure 10:**
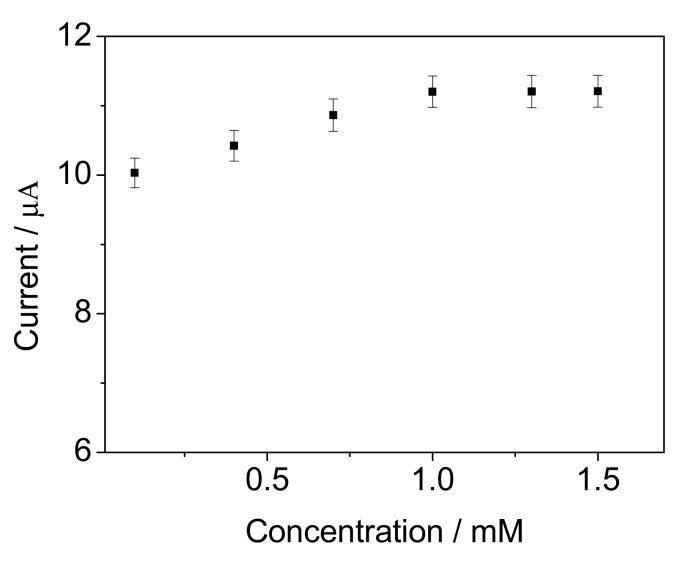
The influence of Py concentration on the current response for 50 mg/L BOD standard solution. The error bars represent three replicate measurements.

The concentration of Py for 3D rGO-PPy preparation is an important influencing factor for immobilizing *B. subtilis* because of the adjustable pore size as mentioned above. We know that the amount of immobilized cells would increase as the pore size of 3D rGO-PPy was enlarged. So the BOD electrochemical signals from the rGO-PPy-*B* microbial biofilms obtained with the Py concentration from 0.1 mM to 1.5 mM were investigated, respectively, by comparing the change of amperometric values (*I*) before and after 15 min incubation in the bioreactor with 50 mg/L BOD at 35 °C. As shown in Figure 10, *I* gradually increased as the Py concentration increased from 0.1 mM to 1.0 mM, but had no obvious increase when the Py concentration continued increasing more than 1.0 mM. The reason was speculated to be that the attached Py monomer on the surface and galleries of rGO sheets approached a saturated value when the Py concentration in GO suspension reached up to 1.0 mM. This has implications for two aspects. On one hand, the pore size of resultant rGO hydrogel was enlarged as the Py concentration increased, but maintained without obvious change when the Py concentration increased more than 1.0 mM. On the other hand, when the Py concentration was up to 1.0 mM, the attached Py content on rGO came to a constant value and the electrodeposited PPy layer would keep unchanged during the same electropolymerization time with the continuous increase of Py concentration in GO suspension. Moreover, at 1 mM Py, the obtained 3D porous rGO-PPy has porediametersofabout 20 µm, which is thought to be ideal for *B. subtilis* cells growth [28]. So 1.0 mM Py is the optimum concentration for 3D porous rGO-PPy preparation to immobilize *B. subtilis* for the resultant rGO-PPy-*B* microbial biofilm.

The cell concentration is another important factor for *B. subtilis* immobilization because the cell concentration is directly related to the amount of immobilized cells. Five different rGO-PPy-*B* microbial biofilms obtained from the cell concentrations of 0.2 × 10^7^ CFU/mL, 1.1 × 10^7^ CFU/mL, 5.4 × 10^7^ CFU/mL, 9.8 × 10^7^ CFU/mL and 19.7 × 10^7^ CFU/mL were, respectively, used for electrochemical measurement of BOD standard solutions with concentrations of 5 mg/L, 10 mg/L and 20 mg/L using chronoamperometry method. As shown in Table 2, the obtained current value from the one with cell concentrations of 19.7 × 10^7^ CFU/mLwas the highest compared with other cell concentrations. The results suggest that the biocatalytic ability is proportional to the immobilized bacterial number on the rGO-PPy-*B* microbial biofilm. However, the analysis of the linear regression equation illustrated in Table 2 suggested that the microbial biofilm obtained from cell concentration of 9.8 × 10^7^ CFU/mL had best linear relationship between the current signals and BOD solutions in low concentration. Considering the goal of satisfying water quality standards for surface water within BOD concentrations from 3 mg/L to 10 mg/L [4], 9.8 × 10^7^ CFU/mL was selected as the *B. subtilis* cell concentration for the rGO-PPy-*B* microbial biofilm preparation.

**Figure 11:**
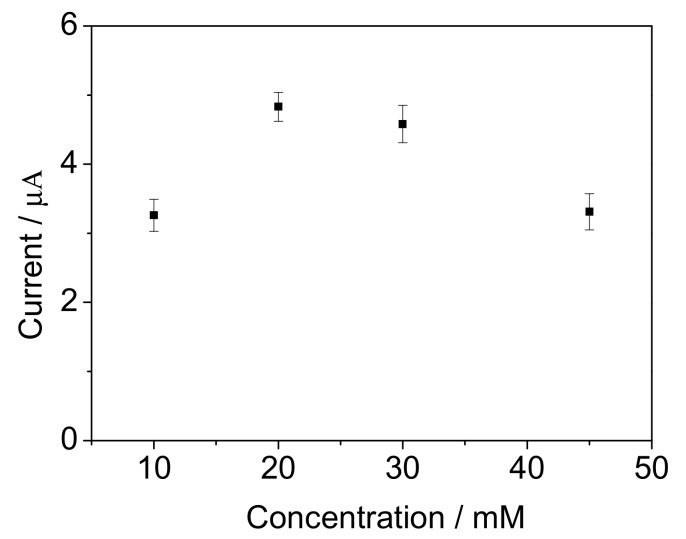
The influence of ferricyanide concentration on the current response for 10 mg/L BOD standard solution. The error bars represent three replicate measurements.

The influence of ferricyanide concentration on the response of the biosensor were evaluated by using 10 mg/L BOD standard solution. Figure 11 shows that the amperometric response reached a high level when the ferricyanide concentration was 20 mM. The response value was obviously reduced when the ferricyanide concentrations were below 10 mM or 45 mM. The reason was speculated to be that the biochemical reaction could be limited by the low concentration of ferricyanide mediator, and the *B. subtilis* viability was suppressed by the toxicity of the mediator when the ferricyanide concentration was above 45 mM.

The incubation time were also investigated by using 10 mg/L BOD solution. The results (data not shown) indicated that the amperometric response increased as the incubation time prolonged from 5 to 60 min. Considering the requirement of fast detection, 15 min was selected as the incubation time for each water sample in subsequent experiment. According to our previous study [26], the incubation pH and temperature was selected as 7.0 and 37 °C, which was in agreement withthe optimum growth condition of *B. subtilis* bacteria.

### 3.5. Performance of the Mediated BOD Biosensor 

**Figure 12:**
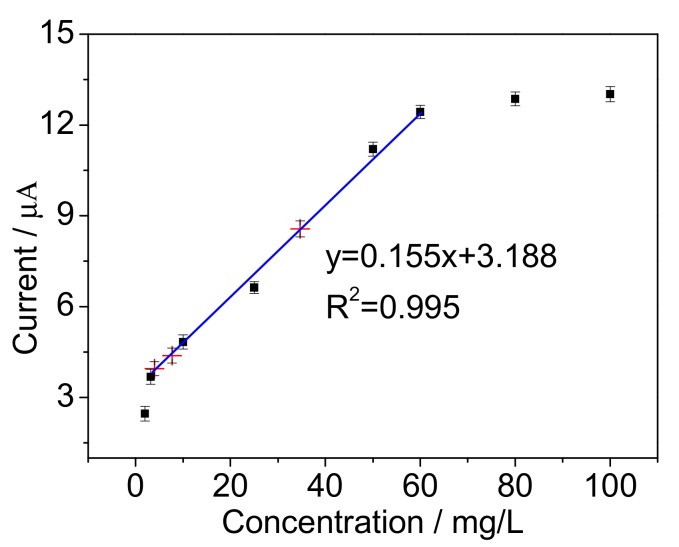
The calibration curve of the mediated BOD biosensor. The red intersection points “×” standards for BOD measurement of real water samples. The error bars represent three replicate measurements.

The linear range of the proposed BOD biosensor was further studied. A response range was evaluated with low concentrations of BOD standard solutions from 2 mg/L to 100 mg/L BOD. Figure 12 shows a linear correlation between amperometric responses and BOD concentrations from 4 to 60 mg/L, and the linear regression equation was *y* = 0.155*x* + 3.188, where *y* is current response and *x* is the concentration of BOD standard solution. The limit of detection as 1.8 mg/L was calculated (S/N ≥ 3). The result revealed that the bacterial degradation efficiency gradually decreased with increasing the organic substrate concentration. The measurement of real water was also carried out using the proposed BOD biosensor. Three real water samples were measured and BOD values of 3.2 mg/L, 7.7 mg/L and 34.7 mg/L (the red intersection points “×” shown in Figure 12) were calculated from their current responses according to the calibration curve. Compared with the third organization data of 4.1 mg/L, 8.6 mg/L and 38.3 mg/L using BOD_5_ method, the error values of 12.6%, 10.2% and 9.3% for BOD measurement were acceptable. Thus, the experimental results indicate that the proposed BOD biosensor is usable and holds a potential practical application of real water monitoring.

**Figure 13:**
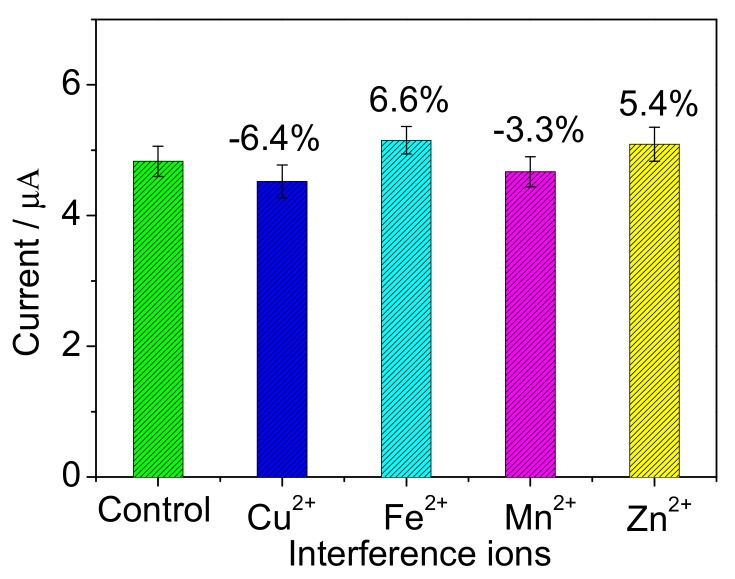
Interference of some heavy metal ions on the biosensor response to 10 mg/L BOD. The error bars represent three replicate measurements.

**Figure 14:**
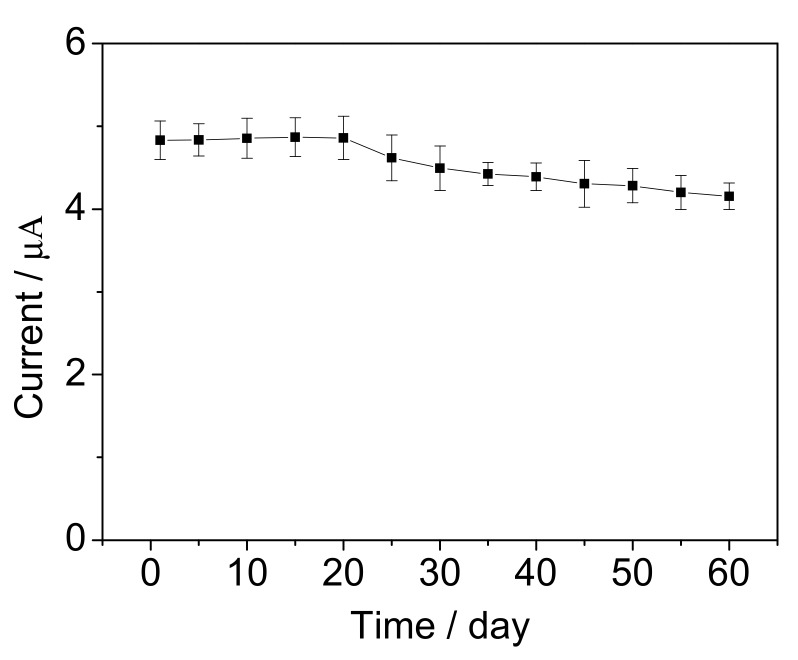
Long-term stability of the BOD biosensor. The error bars represent three replicate measurements.

Some heavy metal ions, such as Cu^2+^, Zn^2+^, Mn^2+^ and Fe^3+^, are common in the surface water and may disturb the biodegradation activity of bacteria cells [39]. Therefore, the influences of these heavy metal ions were investigated. The BOD concentration of 10 mg/L was used as control, and 0.5 mg/L each heavy metal ion was added before measurement. As illustrated in Figure 13, compared with control solution, the response current increased or decreased by less than 10%, respectively. It suggests that the proposed biosensor has good anti-interference ability to the heavy metal ions of Cu^2+^, Zn^2+^, Mn^2+^ and Fe^3+^. In further, long-term stability of the biosensor was investigated by measuring 10 mg/L BOD. As shown in Figure 14, the signal remained 86% after preservation of the microbial biofilm for 60 d. The signal was enhanced in the first 20 d, which was possibly because the bacteria divided continually and some new bacteria reproduced. In the following days, the signal dropped slowly, the reason of which was surmised that the bacteria stopped growing and the cells came into decline stage.

For comparison, the analytical performance of the proposed biosensor and some other mediated BOD biosensors were listed in Table 3. Under the short response time of 15 min, the wide linear range of 4–60 mg/L with a low detection of 1.8 mg/L was obtained, which not only meets the detection standards of I~III classed water quality for domestic wastewater [5], but also satisfies the detection standards of I~V classed water for surface water [4]. The results imply that the 3D porous structure of the rGO-PPy-*B* can provide more space for the biochemical reaction between bacteria and organic matter, leading to an improved degradation efficiency. The long-term stability of 60 d is obvious longer than other mediated biosensors based on biofilm modified electrodes, which suggests that the separation of microbial film and working electrode can avoid sensor fouling from frequent cleaning and recalibration [26]. The reason of the stability being shorter than the sensor reported in reference of [40] is speculated that the bacteria isolated from real water could possibly have stronger bioactivity and anti-poisoning ability. However, the lower linear range value of 4 mg/L much lower than the reported sensor of [41] indicates that pure bacteria could proper degradation of organic matter in low concentration. 

## 4. Conclusions

We developed a mediated BOD biosensor for BOD measurement in low concentration. The major innovation is that 3D porous rGO-PPy composite was synthesized for *B. subtilis* immobilization. On one hand, the hydrothermally prepared rGO-PPy hydrogel has a 3D porous microstructure which can enlarge the effective specific surface area for bacteria immobilization, and the maintained some functional groups such as carboxyl, hydroxyl and epoxy on rGO, which can coordinate with the groups of amion and hydroxyl on *B. subtilis*. On the other hand, a positive charged polypyrrole layer was situ-synthesized via electrochemical method enhanced the adsorption ability for negative charged *B. subtilis* immobilization on rGO-PPy composite surface. The experimental results showed that *B. subtilis* had been immobilized on 3D porous rGO-PPy composite surface and maintained a good stability for mediated BOD measurement in low concentration. The present strategy offers a new way for resolving the problem of microorganism cultivation multiple times in mediated BOD biosensors development, and is more useful in practical application for environmental analysis.

## Figures and Tables

**Table 1:** Wet weight of different supports for *B. subtilis* immobilization.

Support	Before Immobilization (mg)	After Immobilization (mg)	ImmobilizedCells (mg)
rGO	20 ± 2	40 ± 7	20 ± 5
rGO-Py	20 ± 2	100 ± 10	80 ± 8
rGO-PPy	20 ± 2	140 ± 12	120± 10

**Table 2:** Comparison of the relationship between cell concentration for microbial biofilm preparation and BOD measurement in low concentration.

Cell Concentration (CFU/mL)	Current Value for BOD Measurement (µA)			Regression Equation	Linear Correlation
	5 mg/L BOD	10 mg/L BOD	25 mg/L BOD		
0.2 × 10^7^	0.91 ± 0.23	1.02 ± 0.21	2.1 ± 0.21	*y* = 0.083*x* + 0.37	0.938
1.1 × 10^7^	2.01 ± 0.22	2.82 ± 0.14	3.43 ± 0.21	*y* = 0.089*x* + 1.705	0.928
5.4 × 10^7^	2.96 ± 0.19	3.63 ± 0.13	4.32 ± 0.17	*y* = 0.087*x* + 2.615	0.967
9.8 × 10^7^	4.16 ± 0.18	4.83 ± 0.23	5.99 ± 0.19	*y* = 0.122*x* + 3.553	0.998
19.7 × 10^7^	6.03 ± 0.25	6.24 ± 0.27	6.31 ± 0.24	*y* = 0.017*x* ± 5.995	0.794

**Table 3:** Comparison to other mediated BOD approaches from previous reports.

Support	Microorganism	Mediator	Linear Range (mg/L)	Detection Limit (mg/L)	Response Time (min)	Stability	Reference
PVA–SbQ	Bacteria isolated from active sludge	HCF (III)	15–200	--	15	7	[40]
Ormodils-PVA	*E. marius*, *B. horikoshii*, *H. marina*	ferricyanide	1.2–40	0.8	30	1 h	[41]
Carbon fiber felt	Bacteria isolated from waste water	ferricyanide	12–100	--	60	110 d	[42]
No support	*E. coil*	ferricyanide	5–400	--	60	--	[43]
PPy	*P. aeruginosa*	Poly (neutral red)	5–100	3	20	10	[29]
3D porous rGO-PPy	*B. subtilis*	ferricyanide	4–60	1.8	15	60	This study
